# Real-World Effectiveness and Safety of Upadacitinib Versus Abrocitinib in Moderate-to-Severe Atopic Dermatitis: A Retrospective Cohort Study

**DOI:** 10.3390/ph19060828

**Published:** 2026-05-25

**Authors:** Ömer Karakoyun, Erhan Ayhan

**Affiliations:** Department of Dermatology and Venereal Diseases, Faculty of Medicine, Dicle University, 21280 Diyarbakır, Türkiye

**Keywords:** atopic dermatitis, upadacitinib, abrocitinib, JAK1 inhibitors, real-world study, Eczema Area and Severity Index (EASI), pruritus, safety

## Abstract

**Background:** Janus kinase (JAK) inhibitors have expanded the therapeutic landscape for moderate-to-severe atopic dermatitis (AD), yet real-world comparative data on upadacitinib and abrocitinib remain limited. **Objective:** We aimed to compare the 24-week real-world effectiveness and safety of upadacitinib 30 mg and abrocitinib 200 mg in adult patients with moderate-to-severe AD, using EASI-75 as the primary efficacy endpoint, and to explore potential clinical factors associated with treatment response. **Methods:** This retrospective single-center study included adult patients with moderate-to-severe AD treated with upadacitinib 30 mg or abrocitinib 200 mg and followed for 24 weeks in routine clinical practice. A small pediatric subgroup (*n* = 3) receiving abrocitinib 100 mg was analyzed separately for descriptive purposes only. Clinical outcomes included Eczema Area and Severity Index (EASI), Peak Pruritus Numerical Rating Scale (PP-NRS), EASI-50/75/90 response rates, and NRS-4 response. Safety was assessed using routinely documented adverse events. Exploratory analyses evaluated the possible influence of body mass index (BMI), baseline immunoglobulin E (IgE), and psychological stress on treatment response. **Results:** Both upadacitinib and abrocitinib were associated with rapid and sustained clinical improvement over 24 weeks. At month 6, EASI-75 response was 83.3% in the upadacitinib group versus 70.8% in the abrocitinib group (*p* = 0.27). EASI-50 response was significantly higher in the upadacitinib group (100% vs. 83.3%; *p* = 0.05). Exploratory findings suggested that higher BMI, elevated baseline IgE levels, and psychological stress may be associated with less favorable response trajectories. Both treatments demonstrated a generally favorable safety profile; however, one serious cerebrovascular thrombotic event occurred in a patient with pre-existing cardiovascular risk factors, highlighting the importance of individualized risk assessment when prescribing JAK inhibitors. **Conclusions:** In routine clinical practice, both upadacitinib and abrocitinib appeared effective for the management of moderate-to-severe AD over 24 weeks. Although upadacitinib 30 mg showed a numerically higher month-6 EASI-50 response, this finding should be interpreted cautiously given the modest sample size and the absence of broader between-group differences across other key efficacy outcomes. Larger prospective studies are needed to confirm comparative effectiveness and to clarify predictors of response and safety in real-world settings.

## 1. Introduction

Atopic dermatitis (AD) is a chronic, relapsing inflammatory dermatosis characterized by eczematous lesions, intense pruritus, sleep disturbance, and substantial impairment in quality of life [1]. Its pathogenesis is driven by type 2-skewed inflammation, with cytokines such as IL-4, IL-13, and IL-31 contributing to epidermal barrier dysfunction, immune dysregulation, and persistent itch, thereby perpetuating the itch–scratch cycle [2,3,4]. Many of the key cytokines implicated in AD signal through the Janus kinase–signal transducer and activator of transcription (JAK–STAT) pathway, making this axis a rational therapeutic target [5]. In particular, IL-4 and IL-13 signaling involves JAK-dependent pathways, including JAK1-containing signaling complexes, which activate downstream STAT programs and amplify inflammatory and barrier-disrupting effects [3,6]. IL-31, a central mediator of pruritus in AD, can also activate neuronal itch pathways through JAK–STAT signaling mechanisms [2,4,7].

The introduction of oral selective JAK1 inhibitors has expanded systemic treatment options for moderate-to-severe AD by offering rapid itch relief together with clinically meaningful improvements in skin inflammation [5,8,9]. In the pivotal phase 3 Measure Up 1 and Measure Up 2 trials, upadacitinib demonstrated significant efficacy versus placebo in adolescents and adults with moderate-to-severe AD, with substantial improvements in both disease severity and pruritus and an acceptable safety profile [8,9]. Similarly, abrocitinib showed significant efficacy as monotherapy and in comparative settings versus placebo and dupilumab, including early antipruritic effects in JADE COMPARE and 26-week comparative efficacy data versus dupilumab in JADE DARE [10,11]. However, randomized controlled trials may not fully capture the complexity of routine clinical care, where treatment responses are influenced by patient heterogeneity, comorbidities, adherence patterns, prior systemic exposure, and individualized therapeutic decision-making [12]. This gap underscores the importance of real-world comparative evidence [12].

In everyday practice, the choice between oral JAK1 inhibitors is often shaped not only by expected speed of response and tolerability considerations, but also by patient-level characteristics that may modify treatment outcomes [5,12]. Psychological stress may exacerbate itch perception and scratching behaviors and has been associated with poorer symptom control in AD, highlighting the clinical relevance of psychosocial context [13,14,15]. Baseline total IgE, a marker linked to atopic immune activation, has been associated with disease severity patterns in contemporary clinical datasets and may also be relevant to variability in treatment response [16,17]. Likewise, excess adiposity and higher body mass index (BMI) have been associated with greater AD burden in observational studies and may contribute to systemic inflammatory load, with possible implications for treatment response in real-world settings [18,19]. Despite the increasing use of oral JAK inhibitors, head-to-head real-world comparisons of upadacitinib and abrocitinib with follow-up extending to approximately 24 weeks remain limited [12,20].

Therefore, we conducted a retrospective observational cohort study to compare the 24-week effectiveness and safety of upadacitinib versus abrocitinib in patients with moderate-to-severe AD treated in a tertiary dermatology center and to explore whether BMI, baseline IgE, and psychological stress were associated with less favorable treatment response trajectories [12].

## 2. Results

### 2.1. Patient Characteristics and Treatment Distribution

**Figure 1:**
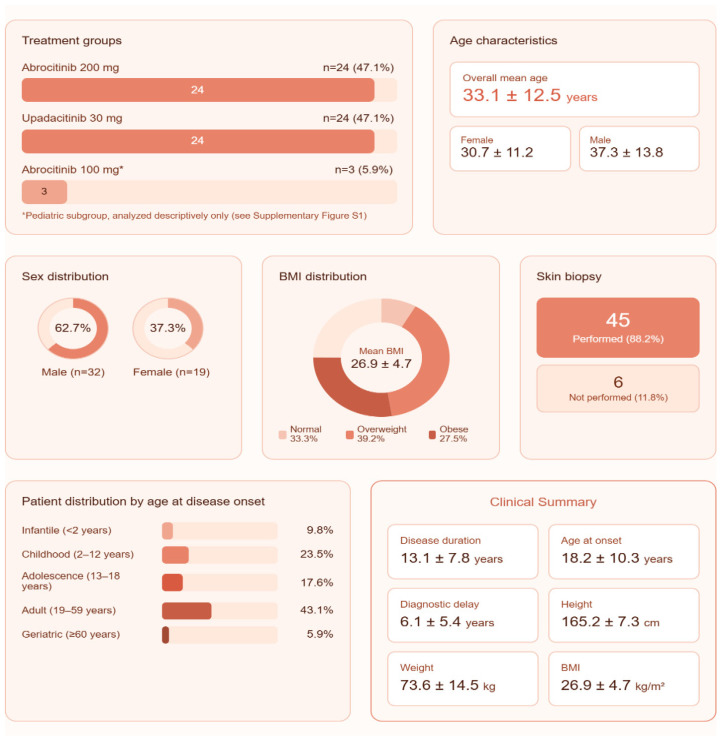
Flow of patient inclusion and treatment distribution in the study cohort.

A total of 51 patients were included in the study. Of these, 62.7% (n = 32) were male and 37.3% (n = 19) were female, with a mean age of 33.1 ± 12.5 years and a mean height of 165.2 ± 7.3 cm. The mean disease duration was 13.1 ± 7.8 years, and the mean diagnostic delay was 6.1 ± 5.4 years. Regarding age at disease onset, adult-onset AD (19–59 years) constituted the largest subgroup (43.1%). In terms of body mass index, 33.3% of patients were normal weight, 39.2% were overweight, and 27.5% were obese. The main adult treatment groups were upadacitinib 30 mg (n = 24; 47.1%) and abrocitinib 200 mg (n = 24; 47.1%). A small pediatric subgroup received abrocitinib 100 mg (n = 3; 5.9%) and was analyzed separately (Figure 1).

### 2.2. Prognostic and Laboratory Parameters

**Figure 2:**
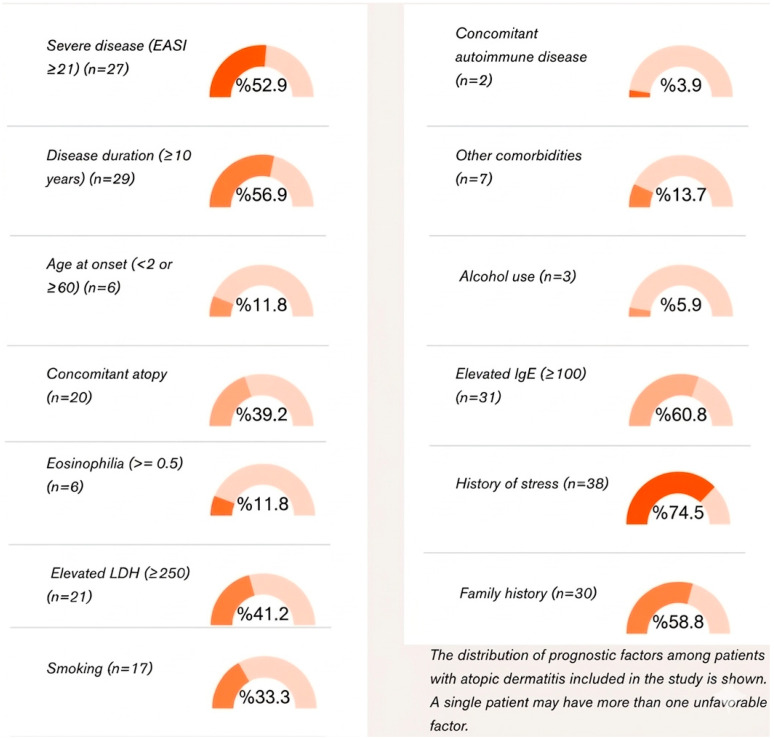
Baseline prognostic and laboratory characteristics of the study population.

At baseline, 52.9% of patients were classified as having severe AD (EASI ≥ 21), and 39.2% had concomitant atopy. A positive family history was present in 58.8%. A history of psychological stress, assessed via structured clinical interview as patient-reported significant stress exposure (including work-related stress, family conflicts, financial difficulties, or major life events) within the preceding 6 months, was reported by 74.5% of patients. On baseline laboratory assessment, the most frequent abnormalities were elevated total IgE (>100 IU/mL; 60.8%) and elevated LDH (≥250 U/L; 41.2%) (Figure 2).

### 2.3. Prior Treatment History

**Figure 3:**
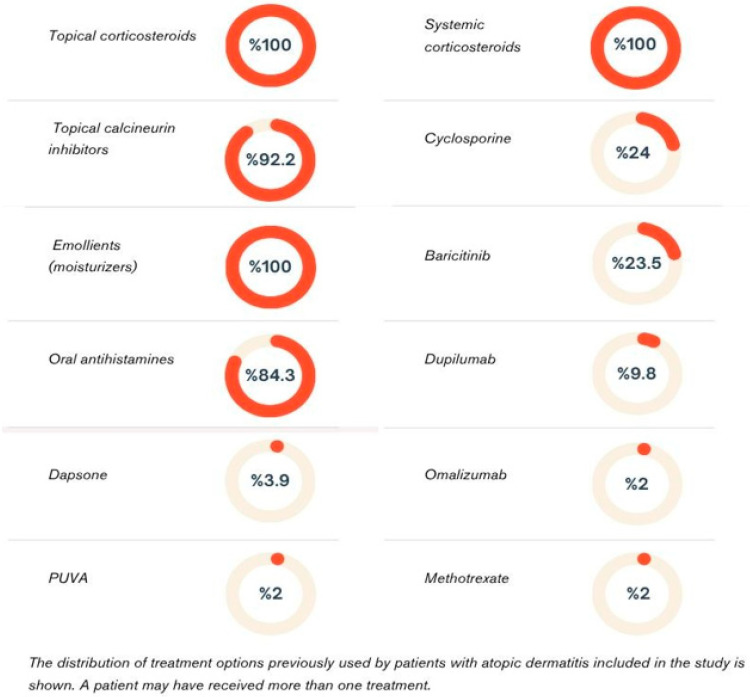
Previous treatment history of patients before initiation of selective JAK1 inhibitor therapy.

All patients had previously used topical corticosteroids, emollients, and systemic corticosteroids (100%). Use of topical calcineurin inhibitors (92.2%) and oral antihistamines (84.3%) was also common. Prior exposure to systemic agents included cyclosporine (24.0%), baricitinib (23.5%), and dupilumab (9.8%). Among dupilumab-experienced patients (n = 5), reasons for switching to JAK inhibitor therapy included inadequate response (n = 3) and tolerability issues (n = 2) (Figure 3).

### 2.4. Effectiveness Outcomes

**Figure 4:**
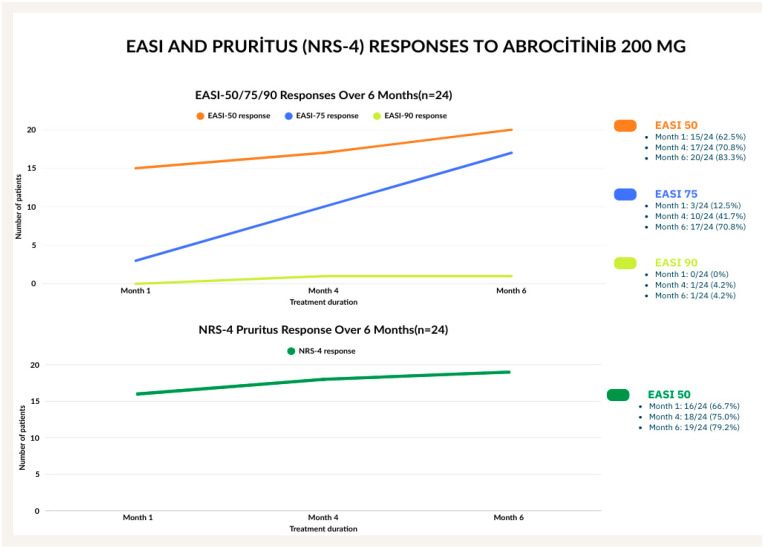
Longitudinal effectiveness outcomes in the abrocitinib 200 mg group over 24 weeks.

In the abrocitinib 200 mg group, the EASI-50 response rate increased from 62.5% at month 1 to 70.8% at month 4 and 83.3% at month 6. The EASI-75 response rate rose from 12.5% at month 1 to 70.8% at month 6, whereas EASI-90 response remained low, with only a limited increase during follow-up. The pruritus response rate (NRS-4) at month 6 was 79.2% (Figure 4).

**Figure 5:**
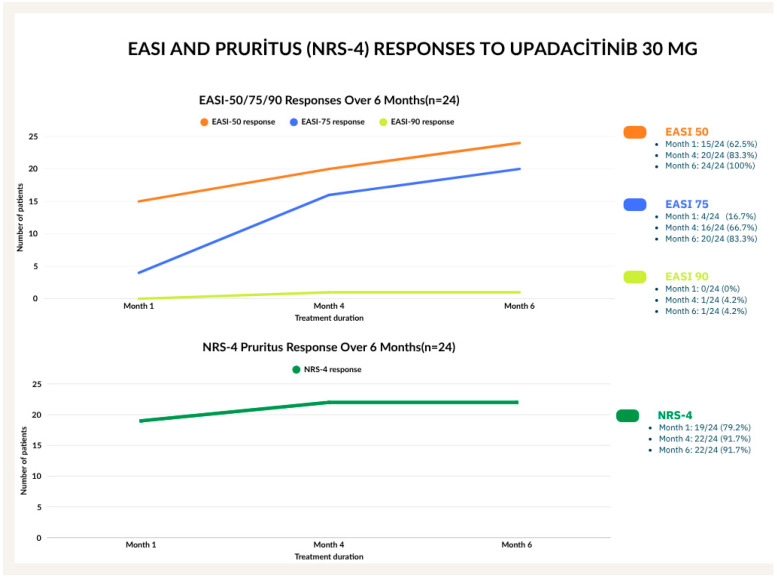
Longitudinal effectiveness outcomes in the upadacitinib 30 mg group over 24 weeks.

In the upadacitinib 30 mg group, response rates increased more markedly over time. EASI-50 reached 100% by month 6, and NRS-4 response reached 91.7% at months 4 and 6. The EASI-75 response rate at month 6 was 83.3%, whereas EASI-90 remained low in both adult treatment groups (Figure 5).

The pediatric subgroup receiving abrocitinib 100 mg (n = 3) was not included in the main comparative analyses due to the very small sample size, which precludes meaningful statistical inference. Descriptive results for this subgroup are presented in Supplementary Figure S1.

### 2.5. Comparative Analyses (Upadacitinib 30 mg vs. Abrocitinib 200 mg)

Comparative response rates between the two adult treatment groups at months 1, 4, and 6 are summarized in Table 1, and corresponding trends are illustrated in Figure 4 and Figure 5. At month 6, the primary endpoint EASI-75 response was achieved by 83.3% of patients in the upadacitinib group compared with 70.8% in the abrocitinib group; although numerically higher in the upadacitinib group, this difference was not statistically significant (*p* = 0.27). For the secondary endpoint EASI-50, response rates at month 1 were identical in both groups (62.5% vs. 62.5%; *p* = 1.00). By month 6, EASI-50 response was 100% in the upadacitinib group compared with 83.3% in the abrocitinib group, reaching borderline statistical significance (*p* = 0.05). EASI-90 response remained limited in both groups (4.2% in each group at month 6; *p* = 1.00). For pruritus control (NRS-4), the upadacitinib group showed numerically higher response rates at months 4 and 6 (91.7%) than the abrocitinib group (75.0% and 79.2%, respectively), although these differences were not statistically significant (month 6: *p* = 0.26).

### 2.6. Factors Associated with Treatment Response

Sex and age were not significantly associated with treatment response (*p* > 0.05). In contrast, obesity (*p* = 0.04), elevated baseline total IgE (*p* = 0.04), and a history of psychological stress (*p* = 0.03) were associated with lower EASI-75 response rates. Elevated LDH showed a borderline negative association with EASI-90 response (*p* = 0.05). Patients reporting stress also more frequently reported subjective complaints such as sleep disturbance and headache (*p* = 0.05) (Figure 2).

### 2.7. Safety

**Figure 6:**
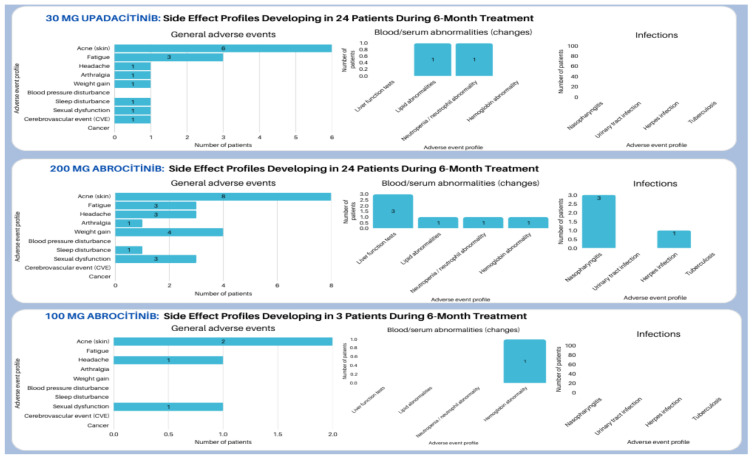
Adverse events and safety profile in patients.

The most commonly reported clinical adverse event was acne in both adult treatment groups (upadacitinib, 25.0%; abrocitinib, 33.3%). In the abrocitinib 200 mg group, acne and weight gain tended to occur more frequently; three patients developed elevated liver function test results, and one patient experienced a herpes infection. In the upadacitinib group, one patient experienced a cerebrovascular thrombotic event. This patient was 69 years old and had a history of hypertension, but had no history of smoking, alcohol use, prior thrombotic events, or known family history of thromboembolic disease. Overall, no statistically significant between-group difference in adverse event profiles was observed (*p* > 0.05) (Figure 6).

## 3. Discussion

In this retrospective real-world cohort, both upadacitinib and abrocitinib were associated with rapid and sustained improvement in disease severity and pruritus over 24 weeks in patients with moderate-to-severe AD. At month 6, upadacitinib achieved numerically higher response rates than abrocitinib for EASI-50 (100% vs. 83.3%; *p* = 0.05) and EASI-75 (83.3% vs. 70.8%; *p* = 0.27), while improvement in pruritus (NRS-4) was also numerically greater with upadacitinib (91.7% vs. 79.2%; *p* = 0.26). Nevertheless, stringent clearance-level responses (EASI-90) remained low in both adult groups (4.2%), which may reflect the severe, treatment-experienced, and inherently heterogeneous profile of patients managed in a tertiary referral setting [21].

Although AD is often reported to be more prevalent in adult women, higher prevalence among older men and sex-related variation across disease phenotypes have also been described [22]. The male predominance observed in our cohort may therefore reflect referral bias toward patients with more severe, refractory, or long-standing disease, as is often seen in tertiary-care real-world series [21]. This interpretation is further supported by the high proportion of patients with severe baseline disease (EASI ≥ 21), which may also help explain why real-world outcomes can differ from those reported in randomized controlled trials involving more selected populations and more standardized follow-up protocols [21,23].

Our findings also suggest that obesity, elevated baseline total IgE, and psychological stress may be associated with a lower likelihood of achieving EASI-75. Obesity may contribute to systemic inflammatory burden and epidermal barrier dysfunction, thereby aggravating disease severity and potentially attenuating treatment responsiveness [24,25]. Moreover, obesity may amplify pruritus–sleep disturbance cycles and further impair quality of life [26,27], which could indirectly limit therapeutic improvement. Elevated total IgE, reflecting heightened atopic immune activation, has likewise been associated with more severe phenotypes and less favorable outcomes in biomarker-oriented studies [28,29]. Similarly, higher LDH, an indirect marker of inflammatory burden, showed a borderline negative association with stringent response (EASI-90), supporting the concept that greater baseline inflammatory activity may require longer treatment duration or more sustained disease control to achieve deeper response thresholds [30].

Psychological stress was highly prevalent in our cohort and was associated with lower EASI-75 achievement, in keeping with the well-recognized psychoneuroimmunologic interplay in AD, whereby stress may intensify itch perception, scratching behavior, and sleep disruption [26,31]. Taken together, these observations suggest that optimizing outcomes with JAK1 inhibitors in routine practice may require a broader management approach that addresses psychosocial stressors alongside pharmacologic therapy.

The magnitude and trajectory of response observed with upadacitinib are broadly consistent with real-world reports demonstrating high rates of EASI-75 over longer follow-up periods [32,33], while the rapid and sustained antipruritic effect seen in our cohort is in line with the established antipruritic profile of JAK1 inhibition [34]. Responses observed with abrocitinib were likewise broadly comparable to those reported in pivotal trials and extension studies with respect to EASI-75 and itch improvement over time [34,35], supporting its effectiveness in routine clinical care. Although between-group differences did not reach statistical significance for most endpoints, the overall direction of effect suggested a trend toward higher response rates with upadacitinib by month 6. Such variability across cohorts has also been described in retrospective comparative studies and network meta-analyses, in which patient selection, baseline severity, prior treatment exposure, and follow-up intensity may substantially influence observed response patterns [36,37].

Another notable observation in our cohort was the low EASI-90 rate at 24 weeks. This likely reflects the demanding nature of this endpoint in a real-world population characterized by severe, treatment-experienced disease, and it is possible that deeper responses may emerge with longer follow-up, as suggested by previous reports [38,39]. This finding reinforces the notion that achieving deep response may require extended treatment duration together with careful management of factors that perpetuate inflammation and itch.

Overall, both agents were generally well tolerated, and acne was the most common adverse event, consistent with previous reports suggesting an increased frequency of acne during JAK inhibitor therapy [40,41]. Laboratory abnormalities, including lipid alterations, liver function test elevations, and hematologic fluctuations, appeared more prominent in the abrocitinib group, which is broadly consistent with known monitoring considerations from phase 3 programs [42]. Notably, one cerebrovascular thrombotic event occurred in the upadacitinib arm. This patient was a 69-year-old male with pre-existing hypertension, which represents an established cardiovascular risk factor. Although this was an isolated event and no causal inference can be drawn from a single observation in a small retrospective cohort, the occurrence of such a serious adverse event warrants careful consideration. This finding aligns with post-marketing safety signals and regulatory warnings from both the FDA and EMA emphasizing the increased risk of major adverse cardiovascular events (MACE) and venous thromboembolism (VTE) associated with JAK inhibitors, particularly in patients aged ≥65 years or those with cardiovascular risk factors [39]. Consequently, the characterization of the overall safety profile as “favorable” should be interpreted with appropriate caution, and these findings underscore the critical importance of thorough baseline cardiovascular risk assessment, careful patient selection, and vigilant monitoring throughout JAK inhibitor therapy.

## 4. Materials and Methods

### 4.1. Study Design and Ethics

This retrospective cohort study was conducted at the Department of Dermatology and Venereology, Dicle University Faculty of Medicine. The study protocol was approved by the Dicle University Non-Interventional Research Ethics Committee (17 September 2025; approval no. 310) and was conducted in accordance with the Declaration of Helsinki.

### 4.2. Setting, Participants, and Eligibility Criteria

Adult patients aged 18–90 years who attended our outpatient clinic between 1 January 2024 and 15 August 2025 with a diagnosis of moderate-to-severe atopic dermatitis (AD) and who initiated treatment with a selective JAK1 inhibitor were eligible for inclusion. Patients received either upadacitinib 30 mg or abrocitinib 200 mg as part of routine clinical care. A small pediatric subgroup (n = 3) receiving abrocitinib 100 mg was included for descriptive analysis only and was not part of the main comparative analyses.

To be included, patients were required to have at least one post-baseline follow-up visit. Exclusion criteria were substantial missing data, treatment with another systemic or biologic agent within the preceding 4 weeks, and low treatment adherence based on routine clinical documentation.

### 4.3. Data Sources and Collected Variables

Data were retrospectively extracted from the Hospital Information Management System and patient medical records. The following variables were recorded: demographic characteristics (age, sex, and body mass index), clinical characteristics (disease duration and concomitant atopic or systemic comorbidities), and treatment-related information (treatment agent, dose, and treatment initiation date).

### 4.4. Outcomes

Effectiveness was assessed at baseline (month 0) and at months 1, 4, and 6 using the Eczema Area and Severity Index (EASI) and the Peak Pruritus Numerical Rating Scale (PP-NRS). EASI response thresholds were defined as EASI-50, EASI-75, and EASI-90, corresponding to at least 50%, 75%, and 90% improvement from baseline, respectively. Clinically meaningful improvement in pruritus was defined as a reduction of at least 4 points from baseline on the PP-NRS (NRS-4).

The primary effectiveness outcome was the proportion of adult patients achieving EASI-75 at month 6, a clinically meaningful benchmark aligned with current regulatory and clinical trial standards for JAK inhibitor studies in atopic dermatitis. Secondary effectiveness outcomes included EASI-50, EASI-90, and NRS-4 response rates at months 1, 4, and 6.

Safety outcomes included adverse events, laboratory abnormalities, and reasons for treatment discontinuation during follow-up. Safety data were extracted from visit notes and laboratory records and were recorded in a standardized manner.

### 4.5. Statistical Analysis

All statistical analyses were performed using IBM SPSS Statistics, version 21.0 (IBM Corp., Armonk, NY, USA). The distribution of continuous variables was assessed using the Kolmogorov–Smirnov test. Continuous variables are presented as mean ± standard deviation or median (minimum-maximum), with coefficient of variation (CV%) and 95% confidence intervals reported where appropriate.

Within-group changes over time were evaluated using the paired-samples *t* test for normally distributed paired data and the Wilcoxon signed-rank test for non-normally distributed paired data. Correlations were assessed using Pearson’s or Spearman’s correlation coefficients, as appropriate. Categorical variables were compared using Pearson’s chi-square test or Yates’ continuity correction, and Fisher’s exact test was used when expected cell counts were small. Given the small sample size and exploratory nature of some analyses, results with borderline significance (*p* = 0.05) should be interpreted with caution. All statistical tests were two-sided, and a *p* value of <0.05 was considered statistically significant.

## 5. Limitations and Implications

This study has several limitations that warrant careful consideration. First, the retrospective design inherently limits causal inference and introduces potential selection and information biases. Second, the relatively small sample size (n = 24 per adult treatment group) substantially reduces statistical power to detect between-group differences, particularly for secondary endpoints; this limitation is reflected in several numerically different but statistically non-significant comparisons, such as the EASI-75 response rates (83.3% vs. 70.8%; *p* = 0.27), which may represent clinically meaningful differences that our study was underpowered to detect. Third, several *p*-values approached borderline significance (*p* = 0.05), and given the multiple comparisons performed without formal correction, some findings may be susceptible to type I error and should be interpreted cautiously. Fourth, the assessment of psychological stress relied on patient self-report during structured clinical interviews rather than validated psychometric instruments (e.g., Perceived Stress Scale), which limits the precision and reproducibility of this variable. Fifth, the pediatric subgroup (n = 3) was extremely limited and was therefore presented descriptively only in the Supplementary Materials rather than included in comparative analyses. Despite these limitations, our findings contribute clinically relevant mid-term real-world comparative data suggesting that both upadacitinib and abrocitinib may represent effective therapeutic options for moderate-to-severe AD. Finally, our exploratory results raise the possibility that BMI, serum IgE levels, and perceived stress may influence treatment response trajectories; however, these associations require confirmation in larger, adequately powered, prospectively designed studies.

## 6. Conclusions

In routine clinical practice, both upadacitinib and abrocitinib were associated with rapid and sustained clinical improvement in moderate-to-severe atopic dermatitis over 24 weeks, with high pruritus response rates and meaningful reductions in EASI scores. At month 6, the primary endpoint, EASI-75, was achieved by 83.3% of patients in the upadacitinib group versus 70.8% in the abrocitinib group (*p* = 0.27), while EASI-50 response was significantly higher in the upadacitinib group (100% vs. 83.3%; *p* = 0.05). These findings should be interpreted cautiously given the modest sample size and borderline statistical significance. Exploratory analyses suggested that higher BMI, elevated baseline IgE levels, and psychological stress may be associated with less favorable treatment responses, underscoring the potential value of individualized assessment and holistic disease management alongside targeted therapy. Both treatments demonstrated a generally favorable safety profile; however, the occurrence of a serious cerebrovascular thrombotic event in a patient with pre-existing cardiovascular risk factors highlights the critical importance of careful patient selection, comprehensive baseline cardiovascular risk assessment, and vigilant monitoring when prescribing oral JAK inhibitors.

## Figures and Tables

**Table 1:** Comparative response rates at Months 1, 4, and 6 (EASI-50/75/90 and NRS-4) in patients treated with upadacitinib 30 mg versus abrocitinib 200 mg.

Time Point	Response Criterion	Upadacitinib 30 mg (n = 24)	Abrocitinib 200 mg (n = 24)	*p*-Value
**Month 1**	EASI-50	15/24 (62.5%)	15/24 (62.5%)	1.00
	EASI-75 ★	4/24 (16.7%)	3/24 (12.5%)	0.68
	EASI-90	0/24 (0%)	0/24 (0%)	1.00
	NRS-4	19/24 (79.2%)	16/24 (66.7%)	0.33
**Month 4**	EASI-50	20/24 (83.3%)	17/24 (70.8%)	0.31
	EASI-75 ★	16/24 (66.7%)	10/24 (41.7%)	0.08
	EASI-90	1/24 (4.2%)	1/24 (4.2%)	1.00
	NRS-4	22/24 (91.7%)	18/24 (75.0%)	0.12
**Month 6**	EASI-50	24/24 (100%)	20/24 (83.3%)	0.05 *
	EASI-75 ★	20/24 (83.3%)	17/24 (70.8%)	0.27
	EASI-90	1/24 (4.2%)	1/24 (4.2%)	1.00
	NRS-4	22/24 (91.7%)	19/24 (79.2%)	0.26

★ Primary endpoint. EASI = Eczema Area and Severity Index; NRS-4 = ≥4-point reduction in Peak Pruritus Numerical Rating Scale. * Borderline statistical significance; interpret with caution.

## Data Availability

The datasets generated and/or analyzed during the current study are not publicly available due to patient confidentiality and institutional restrictions but are available from the corresponding author on reasonable request, subject to ethics committee approval and data-sharing agreements where applicable.

## Supplementary Materials

The following supporting information can be downloaded at: https://www.mdpi.com/article/10.3390/ph19060828/s1, Figure S1. Descriptive effectiveness outcomes in the pediatric subgroup receiving abrocitinib 100 mg.

## References

[1] Lugović-Mihić L., Barac E., Tomašević R., Parać E., Zanze L., Ljevar A., Dolački L., Štrajtenberger M. (2024). Atopic Dermatitis-Related Problems in Daily Life, Goals of Therapy and Deciding Factors for Systemic Therapy: A Review. Pharmaceuticals.

[2] Huang I.H., Chung W.H., Wu P.C., Chen C.B. (2022). JAK-STAT signaling pathway in the pathogenesis of atopic dermatitis: An updated review. Front. Immunol..

[3] Shankar A., McAlees J.W., Lewkowich I.P. (2022). Modulation of IL-4/IL-13 cytokine signaling in the context of allergic disease. J. Allergy Clin. Immunol..

[4] Łacwik J., Kraik K., Laska J., Tota M., Sędek Ł., Gomułka K. (2025). IL-31/33 Axis in Atopic Dermatitis. Int. J. Mol. Sci..

[5] Guttman-Yassky E., Irvine A.D., Brunner P.M., Kim B.S., Boguniewicz M., Parmentier J., Platt A.M., Kabashima K. (2023). The role of Janus kinase signaling in the pathology of atopic dermatitis. J. Allergy Clin. Immunol..

[6] Kim K., Kim H., Sung G.Y. (2022). An Interleukin-4 and Interleukin-13 Induced Atopic Dermatitis Human Skin Equivalent Model by a Skin-On-A-Chip. Int. J. Mol. Sci..

[7] Zhang Z., Chang C., Xiao L., Su H., Lyu Y., Zhao J., Chen J., Gou K., Zhou J., Wang C. (2025). The Neuroimmune Axis in Atopic Dermatitis: From Pathogenic Mechanisms to Targeted Neuroimmunotherapy. J. Inflamm. Res..

[8] Guttman-Yassky E., Teixeira H.D., Simpson E.L., Papp K.A., Pangan A.L., Blauvelt A., Thaçi D., Chu C.-Y., Hong H.C.-H., Katoh N. (2021). Once-daily upadacitinib versus placebo in adolescents and adults with moderate-to-severe atopic dermatitis (Measure Up 1 and Measure Up 2): Results from two replicate double-blind, randomised controlled phase 3 trials. Lancet.

[9] Simpson E.L., Papp K.A., Blauvelt A., Chu C.-Y., Hong H.C.-H., Katoh N., Calimlim B.M., Thyssen J.P., Chiou A.S., Bissonnette R. (2022). Efficacy and Safety of Upadacitinib in Patients With Moderate to Severe Atopic Dermatitis: Analysis of Follow-up Data from the Measure Up 1 and Measure Up 2 Randomized Clinical Trials. JAMA Dermatol..

[10] Bieber T., Simpson E.L., Silverberg J.I., Thaçi D., Paul C., Pink A.E., Kataoka Y., Chu C.-Y., DiBonaventura M., Rojo R. (2021). Abrocitinib versus Placebo or Dupilumab for Atopic Dermatitis. N. Engl. J. Med..

[11] Reich K., Thyssen J.P., Blauvelt A., Eyerich K., Soong W., Rice Z.P., Hong H.C.-H., Katoh N., Valenzuela F., DiBonaventura M. (2022). Efficacy and safety of abrocitinib versus dupilumab in adults with moderate-to-severe atopic dermatitis: A randomised, double-blind, multicentre phase 3 trial. Lancet.

[12] Rønnstad A.T.M., Møller A.T., Isufi D., Bunick C.G., Chovatiya R., Nielsen M.-L., Alinaghi F., Thomsen S.F., Vestergaard C., Wollenberg A. (2026). Real-World Evidence of Effectiveness and Safety of Abrocitinib, Baricitinib and Upadacitinib in Atopic Dermatitis: A Systematic Review and Meta-Analysis. Am. J. Clin. Dermatol..

[13] Khalil N.B., Coscarella G., Dhabhar F.S., Yosipovitch G. (2024). A Narrative Review on Stress and Itch: What We Know and What We Would Like to Know. J. Clin. Med..

[14] Zhao Q., Tominaga M., Toyama S., Komiya E., Tobita T., Morita M., Zuo Y., Honda K., Kamata Y., Takamori K. (2024). Effects of Psychological Stress on Spontaneous Itch and Mechanical Alloknesis of Atopic Dermatitis. Acta Derm. Venereol..

[15] Schut C., Munz J., Bermpohl F.M.G., Van Laarhoven A.I., Schmidt J., Evers A.W., Kupfer J. (2026). Effects of Psychological Interventions on Itch, Scratching, and Excoriations: A Systematic Review and Meta-analysis. Acta Derm. Venereol..

[16] Yu X., Li L. (2024). A Multi-centre Analysis of Serum IgE Levels in Atopic Dermatitis. Indian J. Dermatol..

[17] Hagino T., Yoshida M., Hamada R., Saeki H., Fujimoto E., Kanda N. (2024). Predictive factors for responders to upadacitinib treatment in patients with atopic dermatitis. J. Dermatol. Treat..

[18] Sendrea A.M., Cristea S., Salavastru C.M. (2024). Association Between Increased Body Mass Index (BMI) and Atopic Dermatitis in Children Attending a Tertiary Referral Center: A Case-Control Study. Cureus.

[19] Zhang A., Silverberg J.I. (2015). Association of atopic dermatitis with being overweight and obese: A systematic review and metaanalysis. J. Am. Acad. Dermatol..

[20] Gwag H.E., Park M., Park S.Y., Hong N., Heo S.J., Jung H.J., Park M.Y., Choi Y.S., Ahn J. (2026). Real-World Effectiveness and Safety of Abrocitinib in Patients with Atopic Dermatitis: A 16-Week Single-Center Retrospective Cohort Study Compared with Upadacitinib and Baricitinib. Dermatol. Ther..

[21] Kim Y., Seo G., Koopman J.J.E., Yee J. (2025). Real-World Effectiveness and Safety of JAK Inhibitors in Atopic Dermatitis: A Systematic Review and Meta-Analysis. Clin. Exp. Allergy..

[22] Tuttle K.L., Forman J., Beck L.A. (2021). Novel systemic treatments in atopic dermatitis: Are there sex differences?. Int. J. Womens Dermatol..

[23] Alvarenga J.M., Yeung J., Prajapati V., Ribero S., Balato A., Marzano A.V., Cruz M.J., Lopes M.J.P., Lazaridou E., Carrascosa J.-M. (2026). Clinical Trial Eligibility in Atopic Dermatitis: Data from a Large Real-World International Cohort. Dermatol. Ther..

[24] Yang S., Zhu T., Wakefield J.S., Mauro T.M., Elias P.M., Man M.Q. (2023). Link between obesity and atopic dermatitis: Does obesity predispose to atopic dermatitis, or vice versa?. Exp. Dermatol..

[25] Rossi M., Bighetti S., Narcisi A., Costanzo A., Bianco M., Malagoli P., Messina F., Gaiani F., Ferrucci S.M., Marzano A.V. (2025). JAK Inhibitors in Atopic Dermatitis: Does Weight Matter? A Real-World, Nationwide Retrospective Study: IL-AD (Italian Landscape Atopic Dermatitis). Dermatol. Ther..

[26] Mack M.R., Kim B.S. (2018). The Itch-Scratch Cycle: A Neuroimmune Perspective. Trends Immunol..

[27] Traidl S., Hollstein M.M., Kroeger N., Fischer S., Heratizadeh A., Heinrich L., Kind B., Siegels D., Abraham S., Schäfer T. (2025). Obesity is linked to disease severity in moderate to severe atopic dermatitis-Data from the prospective observational TREATgermany registry. J. Eur. Acad. Dermatol. Venereol..

[28] Park J.H., Oh S., Park J., Choi Y., Lee J.H. (2025). Predicting Favorable Conditions for the Determination of Initial Use of Janus Kinase Inhibitors in Patients with Moderate to Severe Atopic Dermatitis. J. Clin. Med..

[29] Moreiras-Arias N., Nieto-Fontarigo J.J., Salgado F.J., González-Vilas D., Paredes-Suárez C., Combo-García E., Rodríguez-Otero C., Flórez Á. (2026). Novel Therapeutic Strategies for Atopic Dermatitis: Biomarker Modulation and Clinical Implications. A Systematic Review. Clin. Rev. Allergy Immunol..

[30] Vittrup I., Thein D., Thomsen S.F., Egeberg A., Thyssen J.P. (2024). Risk Factors that Impact Treatment with Oral Janus Kinase Inhibitors Among Adult Patients with Atopic Dermatitis: A Nationwide Registry Study. Acta Derm. Venereol..

[31] Liu A.W., Gillis J.E., Sumpter T.L., Kaplan D.H. (2023). Neuroimmune interactions in atopic and allergic contact dermatitis. J. Allergy Clin. Immunol..

[32] Chiricozzi A., Ortoncelli M., Schena D., Gori N., Ferrucci S.M., Babino G., Napolitano M., Fargnoli M.C., Stingeni L., Rossi M. (2023). Long-term Effectiveness and Safety of Upadacitinib for Atopic Dermatitis in a Real-world Setting: An Interim Analysis Through 48 Weeks of Observation. Am. J. Clin. Dermatol..

[33] Patruno C., Lauletta G., Pezzolo E., Boccaletti V., Rossi M., Caroppo F., Fortina A.B., Russo F., Cocuroccia B., Bello G.D. (2024). Effectiveness and Safety of Upadacitinib for Adolescents with Atopic Dermatitis in a Real-World Setting. Clin. Drug Investig..

[34] Miot H.A., Criado P.R., de Castro C.C.S., Ianhez M., Talhari C., Ramos P.M. (2023). JAK-STAT pathway inhibitors in dermatology. Bras. Dermatol..

[35] Simpson E.L., Sinclair R., Forman S., Wollenberg A., Aschoff R., Cork M., Bieber T., Thyssen J.P., Yosipovitch G., Flohr C. (2020). Efficacy and safety of abrocitinib in adults and adolescents with moderate-to-severe atopic dermatitis (JADE MONO-1): A multicentre, double-blind, randomised, placebo-controlled, phase 3 trial. Lancet.

[36] Drucker A.M., Lam M., Prieto-Merino D., Malek R., Ellis A.G., Yiu Z.Z.N., Rochwerg B., Di Giorgio S., Arents B.W.M., Mohan T. (2024). Systemic Immunomodulatory Treatments for Atopic Dermatitis: Living Systematic Review and Network Meta-Analysis Update. JAMA Dermatol..

[37] Beard A., Trotter S.C. (2024). JAK 1-3 inhibitors and TYK-2 inhibitors in dermatology: Practical pearls for the primary care physician. J. Fam. Med. Prim. Care.

[38] Abdel-Mageed H.M. (2025). Atopic dermatitis: A comprehensive updated review of this intriguing disease with futuristic insights. Inflammopharmacology.

[39] Kridin K., Kridin S., Mayer E., Sawaed W., Ludwig R.J. (2025). Cardiovascular and thromboembolic risks of JAK inhibitors in atopic dermatitis: A global cohort study. J. Eur. Acad. Dermatol. Venereol..

[40] Schneeweiss M.C., Wyss R., Anand P., Jin Y., Mostaghimi A., Barbieri J.S., Merola J.F., Silverberg J.I., Rosmarin D.M., Glynn R.J. (2026). Risk of Acne in Patients with Atopic Dermatitis Starting a JAK Inhibitor vs Th2 Cytokine Inhibitor. JAMA Dermatol..

[41] Avallone G., Mastorino L., Tavoletti G., Macagno N., Barei F., Schena D., Rossi M., Magnaterra E., Antonelli F., Babino G. (2024). Clinical outcomes and management of JAK inhibitor-associated acne in patients with moderate-to-severe atopic dermatitis undergoing upadacitinib: A multicenter retrospective study. J. Am. Acad. Dermatol..

[42] Kirchhof M.G., Prajapati V.H., Gooderham M., Hong C.-H., Lynde C.W., Maari C., Turchin I., Papp K.A. (2024). Practical Recommendations on Laboratory Monitoring in Patients with Atopic Dermatitis on Oral JAK Inhibitors. Dermatol. Ther..

