# Supplementary material for: Real-World Effectiveness and Safety of Upadacitinib Versus Abrocitinib in Moderate-to-Severe Atopic Dermatitis: A Retrospective Cohort Study
Source: Pharmaceuticals (Basel). 2026 May 25;19(6):828. doi: 10.3390/ph19060828 (PMC13305105; doi:10.3390/ph19060828)

Figure S1. Descriptive effectiveness outcomes in the pediatric subgroup receiving abrocitinib 100 mg.

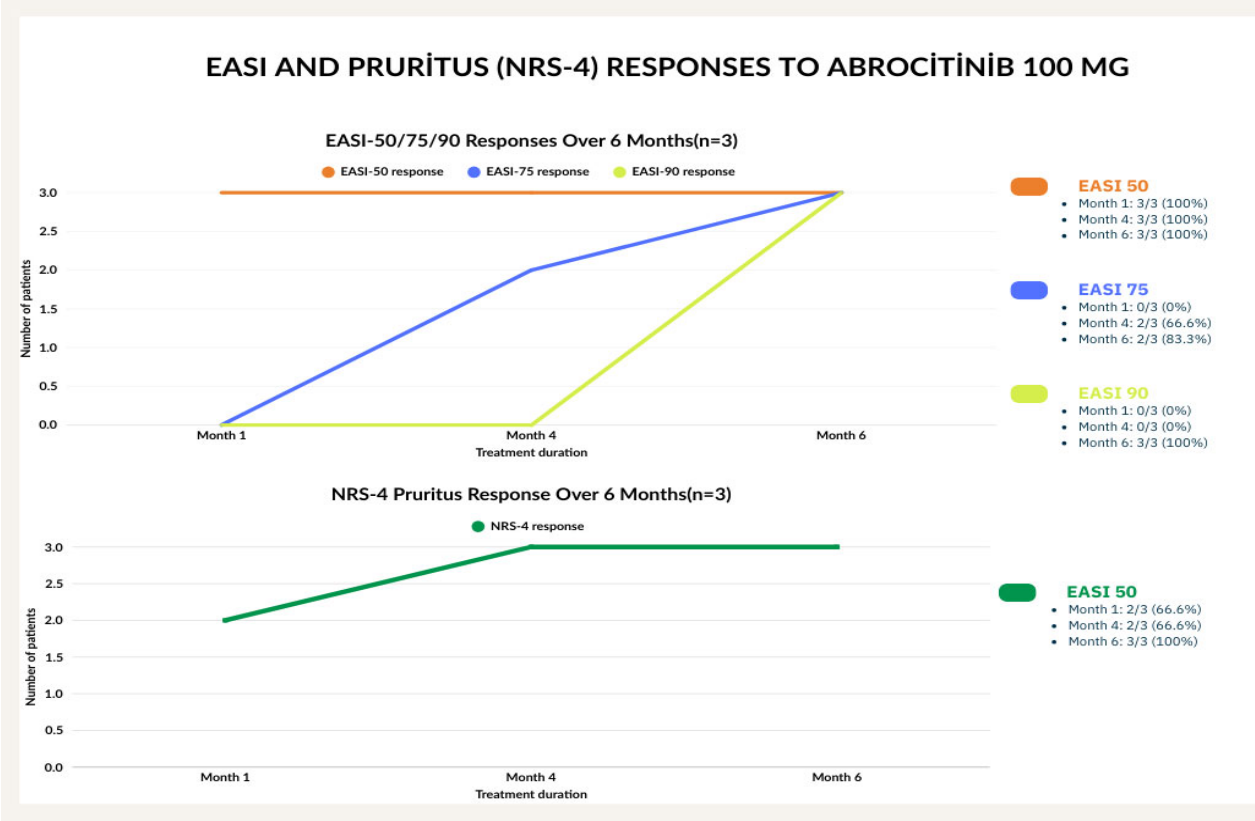

Supplement: Supplementary file 1 [file pharmaceuticals-19-00828-s001.zip › pharmaceuticals-4220143-supplementary.pdf]
